# Multiplane/3D transesophageal echocardiography monitoring to improve the safety and outcome of complex transvenous lead extractions

**DOI:** 10.1111/echo.14318

**Published:** 2019-03-24

**Authors:** Mihai Strachinaru, Chris M. Kievit, Sing C. Yap, Alexander Hirsch, Marcel L. Geleijnse, Tamas Szili‐Torok

**Affiliations:** ^1^ Cardiology Erasmus MC Rotterdam The Netherlands; ^2^ Radiology Erasmus MC Rotterdam The Netherlands

**Keywords:** continuous echocardiography monitoring, intraprocedural echocardiography, multiplane echocardiography, transesophageal echocardiography, transvenous lead extraction

## Abstract

Both transesophageal echocardiography (TEE) and intracardiac echocardiography have been used to assist transvenous lead extractions. The clinical utility of continuous echocardiographic monitoring during the procedure is still debated, with different reports supporting opposite findings. In cases where the procedure is expected to be difficult, we propose adding a continuous TEE monitoring using a static 3D/multiplane probe in mid‐esophageal position, with digital remote manipulation of the field of view. This approach may improve the chances of a successful extraction, increase safety, or even guide the entire intervention. We present here a short case series where continuous monitoring by TEE played an important role.

## 1 BACKGROUND

Both transesophageal echocardiography (TEE) and intracardiac echocardiography have been used to assist transvenous lead extractions (TLE).^1^, ^2^ The clinical utility of continuous echocardiographic monitoring during the procedure is still debated, with different reports supporting opposite findings.^3^, ^4^, ^5^ However, in selected challenging cases, TEE may alter the clinical decision or guide the intervention. We present here a short case series, in typical clinical scenarios, where continuous monitoring by TEE played an important role in the outcome.

## 2 PATIENT 1

**Figure 1:**
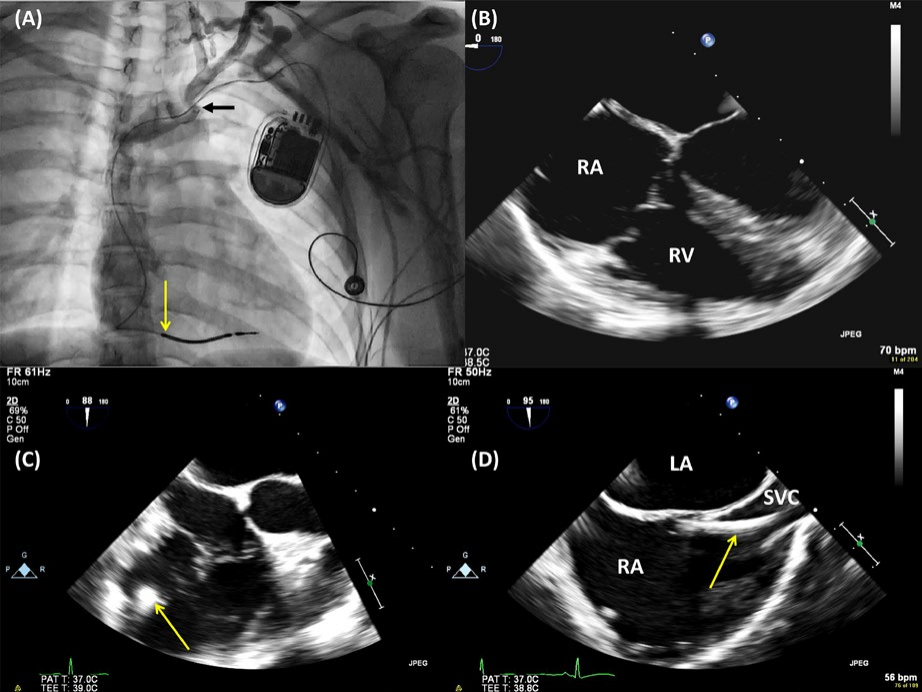
A, Left arm phlebography. The subclavian vein stops abruptly (dark arrow), with collateral filling visible above; the right ventricular lead is indicated with an yellow arrow. B, In 2D transesophageal 4‐chamber view, the lead was difficult to image being situated very posteriorly; C, in short axis of the tricuspid valve, the lead becomes visible; D, bicaval view, demonstrating the relation between the lead and the superior vena cava

**Figure 2:**
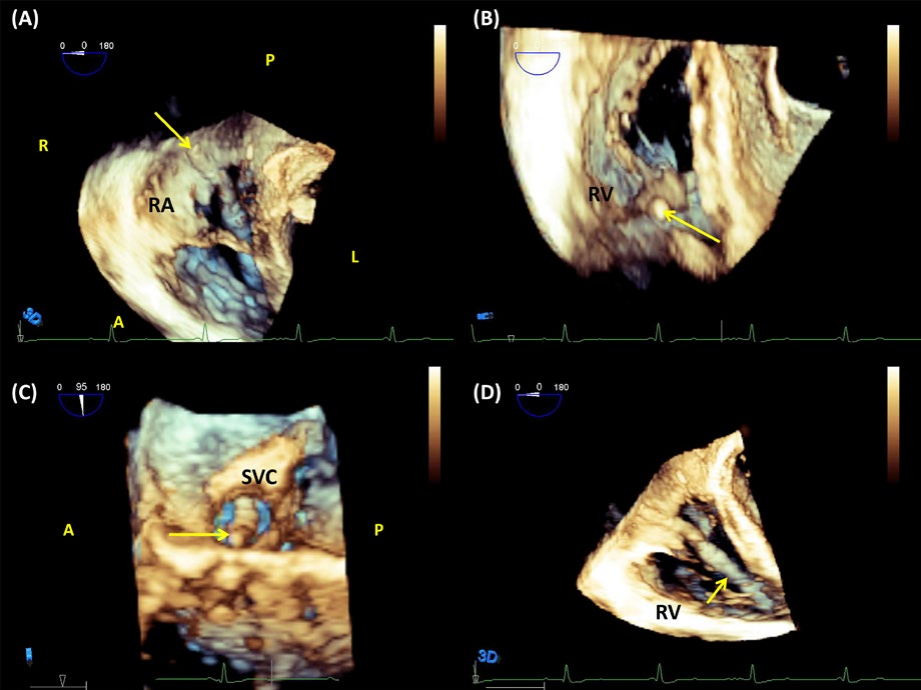
A, On tridimensional echocardiography, the lead was easily identifiable in the posterior commissure of the tricuspid valve from the right atrial aspect of the valve; B, the lead is free from adherences to the valve, coursing further to the apex of the right ventricle (RV). C, Transversal view of the superior vena cava demonstrated a lead (arrow) moving freely from the venous walls; D, RV segment of the lead, visible up to the apex

A 34‐year‐old man had a single‐chamber ICD implanted in 2013 for secondary prevention. In 2017, the shock lead displayed signs of electrical noise resulting in aborted shocks and needed to be replaced. On left arm phlebography, a total occlusion of the left subclavian vein in its mid‐portion was noted, probably in relation to the presence of the lead. Venous return from the left arm followed a collateral supraclavicular system to join the more proximal left subclavian (Figure 1). Because of the venous occlusion, endovascular adhesions were suspected along the lead and we decided to perform the intervention under TEE monitoring. The shock lead was difficult to visualize with 2D echocardiography because of its position in the posteroseptal commissure. Switching to 3D imaging allowed clear visualization of the lead course, from the terminal part of the superior vena cava (SVC), through the tricuspid valve, and up to the right ventricular (RV) apex (Figure 2, Movie S1). No venous or valvular adhesions were noted. Because of a very narrow subclavian passage, but without any venous adhesions, the lead was extracted via the femoral approach using a snare tool (Needle's‐Eye Snare, Cook Medical). A new shock lead was successfully implanted.

## 3 PATIENT 2

**Figure 3:**
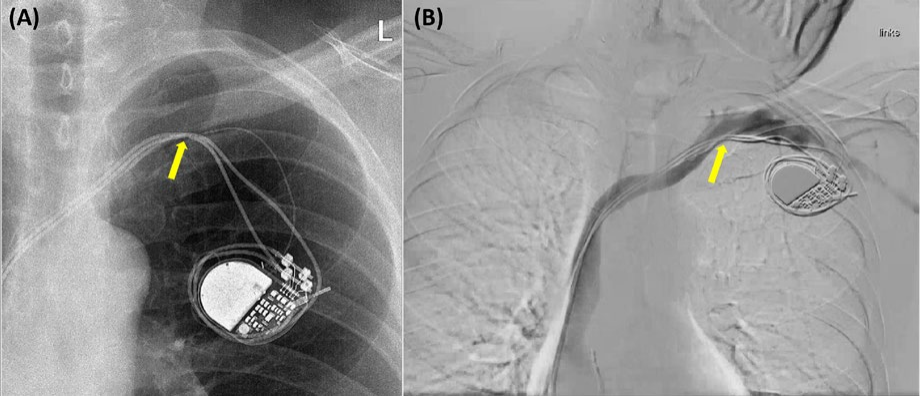
A, crushed leads (arrow) in the subclavian passage, visible on focused X‐ray. Note the presence of three leads (two active leads and one old inactive right ventricular lead); B, left arm phlebography confirming the crush

**Figure 4:**
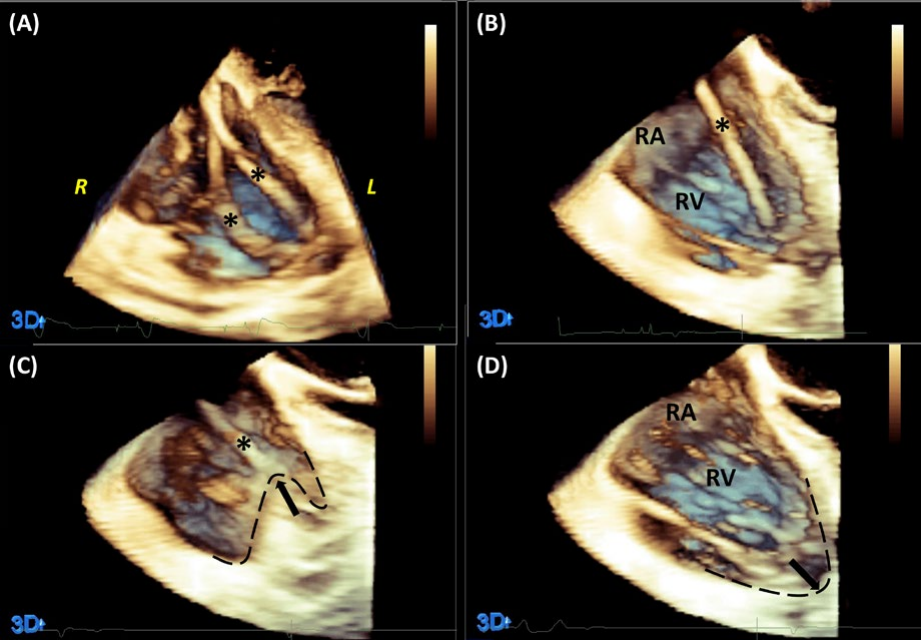
3D echocardiography focused on the right ventricle (RV) and right atrium during the ventricular lead extraction. A, Start of the procedure, with the two RV leads in situ (asterisk). B, The first lead was easily extracted; C, near inversion of the RV cavity (arrow, dashed contour) during pulling of the last lead; D, RV cavity re‐expanded after the lead detached (arrow, dashed contour)

**Figure 5:**
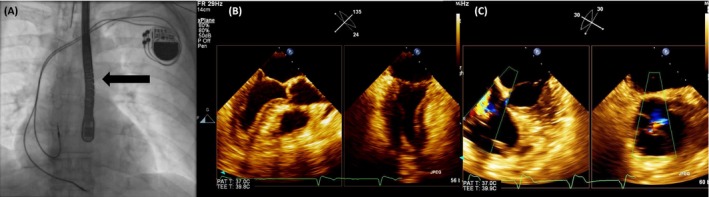
Postimplantation of the new leads. A, Position of the new leads. The static echocardiography probe is also visible in mid‐esophageal position (arrow); B, biplane image of the left cavities; C, biplane image of the tricuspid valve (left long axis, right short axis), displaying a stable pre‐existent tricuspid valve regurgitation. Note that both the left and the right cavities could be imaged in multiplane, by remotely manipulating the field of view, without altering the probe position

A 69‐year‐old man with sick sinus syndrome had a dual‐chamber pacemaker implanted in 2010. Shortly after implantation, both leads became dysfunctional. During the first extraction, both leads were replaced but only the atrial lead could be removed. The old RV lead was abandoned. In 2017, the new leads also became dysfunctional. The chest X‐ray and left arm phlebography showed signs of subclavian crush syndrome (Figure 3). The presence of three relatively old leads was considered to complicate the extraction procedure, which was then performed under continuous TEE monitoring (Figure 4). The locking stylet could not be inserted in the leads (probably due to the subclavian crush); thus, a femoral approach with a snare tool (Needle's‐Eye Snare, Cook Medical) was chosen. The atrial lead was extracted with ease as well as the most recent implanted RV lead. During careful but progressive pulling of the abandoned RV lead, we saw near inversion of the RV cavity with TEE (Figure 4). In this short period, the blood pressure dropped but quickly recovered after the lead detached and the RV re‐expanded (Movie S2). The patient was re‐implanted with a new dual‐chamber pacemaker (Figure 5). No complication was noted, and the patient was quickly discharged.

## 4 PATIENT 3

**Figure 6:**
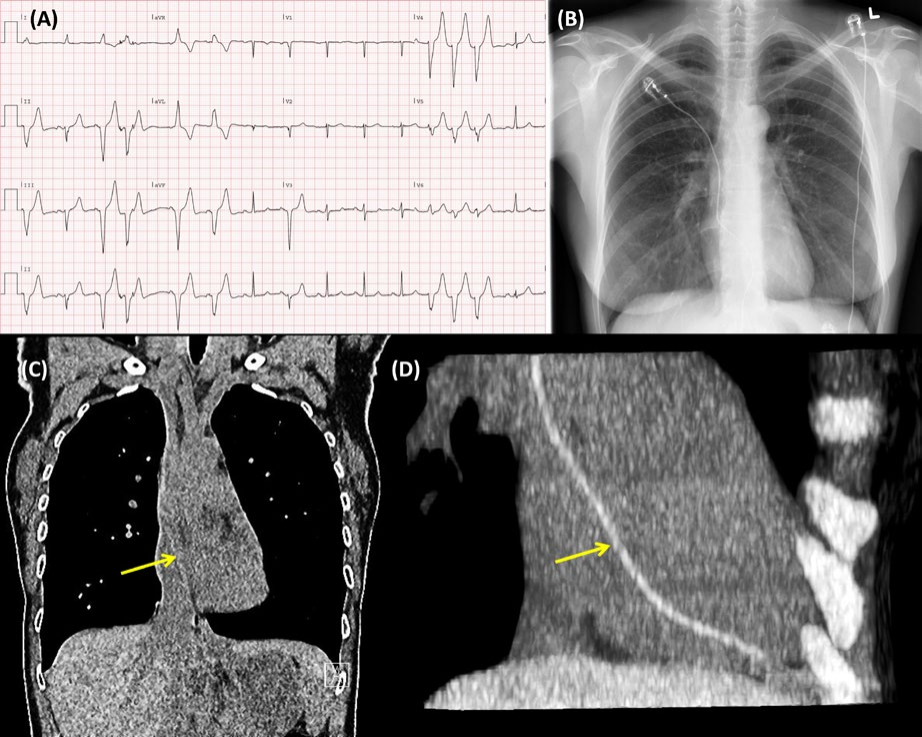
A, ECG sinus rhythm with frequent multifocal premature ventricular complexes; B, normal thorax X‐ray, no lead fragment could be seen; C, CT scan frontal plane, focused on the caval veins: A linear structure (arrow) could be noticed in the proximal superior vena cava. D, CT focused oblique view of the right atrium and right ventricular, demonstrating the linear structure (arrow) coursing to the apex of the right ventricle

**Figure 7:**
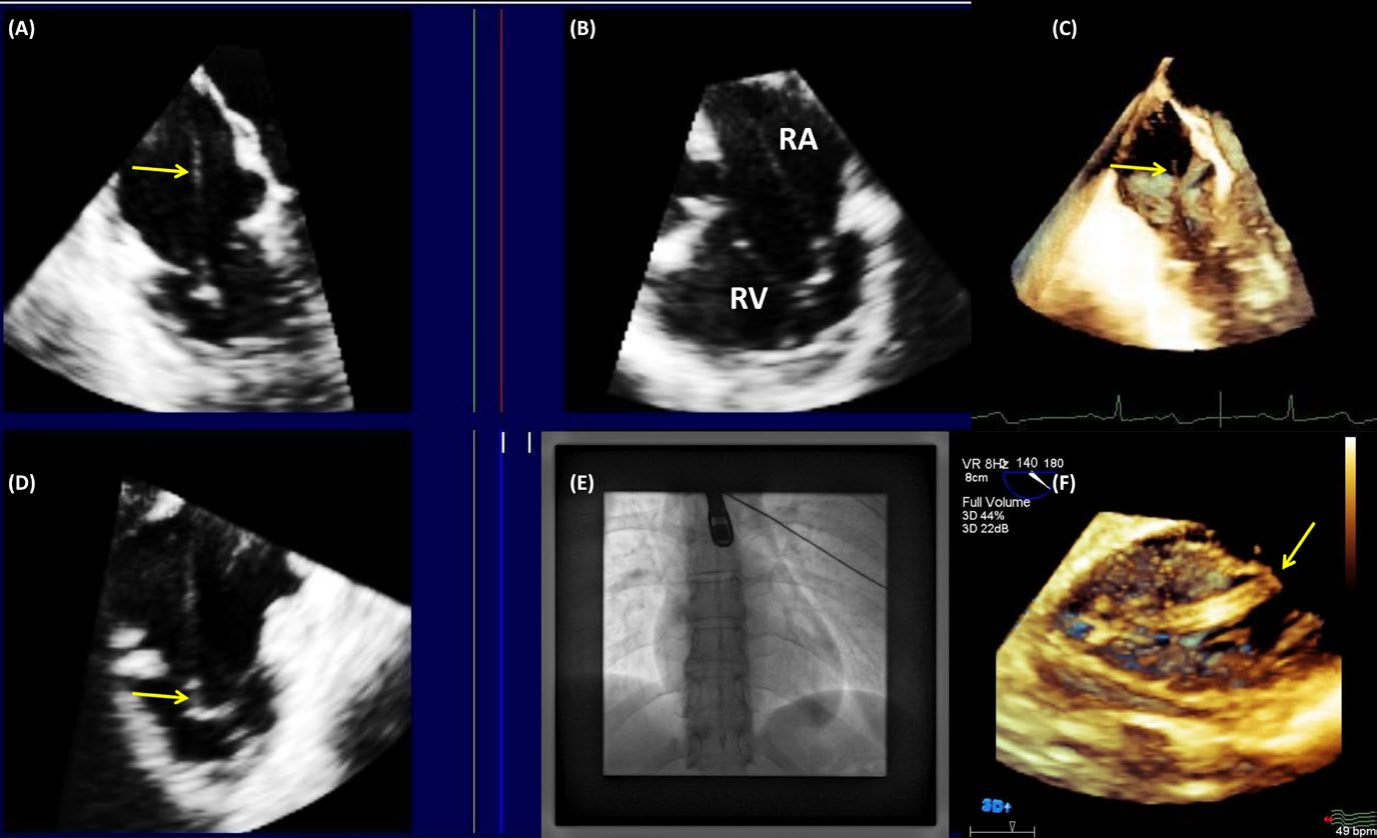
3D and multiplane sections of the right cavities demonstrating the presence of the radiotransparent insulation sheath. A, B, D, triplane sections of right atrium (RA) and right ventricular (RV). The silicone insulation sheath is coursing through the right atrium, the tricuspid valve, and ending in the right ventricular apex (F, arrow); C, 3D view of RA and RV in mid‐esophageal position, the insulation sheath is also visible as an relatively weak but clear linear echo (arrow); E, with high‐resolution angiography, the sheath could not be seen

**Figure 8:**
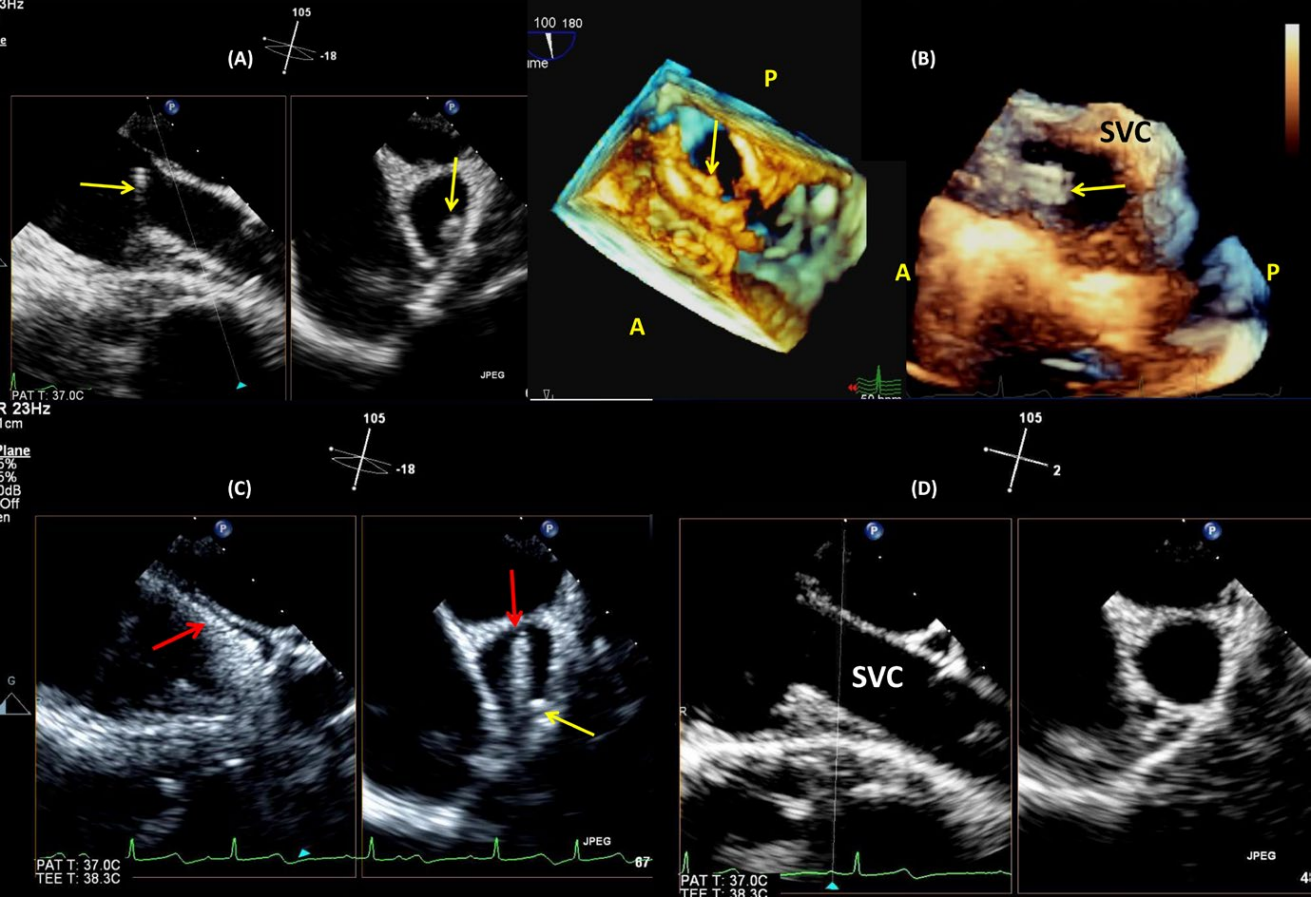
Multiplane/3D echocardiography guiding the extraction. A, biplane image of the superior vena cava (SVC), with the silicone sheath visible (arrow); B, 3D biplane images of the free upper end of the sheath in the last 4 cm of the superior vena cava; C, modified biplane bicaval view: the extraction tool (red arrow) and the free upper end of the sheath (yellow arrow) seen in the superior vena cava; D, following the procedure, the SVC is free of any echos

A 51‐year‐old woman presented to the emergency department with palpitations. One week before, she had undergone a full‐system dual‐chamber pacemaker extraction. No pacemaker was re‐implanted due to the absence of pacing in the previous 5 years. Her ECG at presentation showed sinus rhythm with frequent multifocal premature ventricular complexes and short runs of ventricular tachycardia. Her chest X‐ray was normal and did not demonstrate a complication of the recent lead extraction. On transthoracic echocardiography, a very weak linear echo was visible (Movie S3) in the right atrium (RA). A thoracic CT scan was performed, and a linear structure could be noticed coursing from the proximal SVC to the RV apex (Figure 6). The most probable diagnosis was a retained fragment of the silicone insulation of the previous extracted RV lead. After discussing the case in a heart team, it was decided to try a TEE‐guided percutaneous removal. Surgical removal remained as an escape option. The retained silicone sheath could not be visualized with high‐intensity fluoroscopy; thus, the extraction was guided by TEE. By 3D and multiplane imaging, the silicone insulation sheath was visible from the terminal part of the superior vena cava (Movie S4), coursing through the RA, the tricuspid valve, and ending in the RV apex, “trapped” into the complex trabeculae but without being firmly attached (Figure 7). The upper end of the fragment was found floating freely in the last 4 centimeters of the superior vena cava, clearly visible on multiplane and 3D TEE (Figure 8, Movie S4). A snare tool (Needle's‐Eye Snare, Cook Medical) was guided into the RA using fluoroscopy. Thereafter, guiding was performed using multiplane/3D echocardiography alone, from a modified bicaval view. The snare tool was advanced into the SVC, and the retained silicone sheath was snared and removed (Figure 8, Movie S5). Extraction was complete, confirmed by visual inspection of the insulation fragment and TEE. The patient had no recurrence of ventricular arrhythmia, despite discontinuation of antiarrhythmic drugs. She was quickly discharged to outpatient follow‐up.

## 5 DISCUSSION

Intraprocedural echocardiographic imaging during TLE provides clinically relevant information and is strongly recommended by the current guidelines.^1^

Transesophageal echocardiography can be used to distinguish between free‐floating and adherent leads.^1^, ^2^ Fibrotic adhesions can be present in the subclavian vein, innominate vein, SVC, tricuspid valve apparatus, and RV.^6^ Knowing the sites of fibrotic adhesions may guide the TLE procedure. A contrast venogram of the subclavian vein can identify the presence of stenosis or occlusion in the subclavian and innominate vein, but does not differentiate between free‐floating and adherent leads beyond the SVC. The use of intraprocedural fluoroscopy in combination with manual traction on the lead may give additional information. When cardiac contractility is felt while pulling on the lead, a free‐floating lead is very likely. In the presence of occlusion in the innominate vein (case 1), this maneuver is not useful. In this case, TEE was useful to demonstrate the absence of vascular and valvular adhesions from the level of the SVC to RV. Snaring the lead was deemed the most logical option in this case.

Besides identification of fibrotic adhesions between the trajectory of the SVC and RV, TEE imaging can predict and identify the effect of lead extraction on the tricuspid valve. Knowing the exact relationship between the lead and the tricuspid valve is important to judge the risk of traumatic injury to the tricuspid valve. Case 1 illustrates the added value of 3‐dimensional imaging in comparison with 2‐dimensional TEE imaging in demonstrating the course of the lead across the tricuspid valve. The prevalence of traumatic tricuspid valve injury during lead extraction is variable, ranging from 3.5% to 19%,^1^, ^7^, ^8^, ^9^ and depends on several factors such as the number and type of leads, age, gender, and method of extraction. Significant tricuspid valve regurgitation may lead to or exacerbate heart failure.

Transesophageal echocardiography is also useful for rapidly identifying or excluding cardiovascular causes of hemodynamic instability during a TLE procedure.^3^, ^5^, ^10^ It is especially useful for monitoring pericardial effusion. Another important cause of hemodynamic instability is impairment of venous return during controlled pulling on the leads as demonstrated by case 2. RA or RV retraction limits venous return and lowers LV filling causing hypotension. Hemodynamic stability is acquired by releasing pulling pressure or successful extraction.

Macroscopic inspection of the extracted lead is mandatory to identify missing parts of the leads suggesting retention of lead fragments. Partial lead extraction is relatively rare, with an incidence ranging around 2%–3%.^11^ Usually, distal lead fragments fracture due to mechanical stress during extraction and become obvious on fluoroscopy checkup. Cases have been described of remaining fragments of the silicone protective tube around the lead. These can easily be missed by fluoroscopy if no metallic fragment is retained.^12^, ^13^ Echocardiography however is an ideal imaging tool in this clinical scenario, given the relatively high contrast between intracavitary blood and any type of solid foreign body. This is illustrated by our Patient 3 in whom, although a multimodality approach was needed for the diagnosis, TEE was the only intraprocedural imaging tool allowing successful guidance of the TLE procedure.

Finally, periprocedural TEE is also useful for characterizing lead or valvular vegetations and thrombus formation on externalized cables (eg, Riata leads^1^). Knowing the location and extent of vegetation or thrombus can provide important information to the extractor^14^ (not demonstrated in this case series).

In cases where the procedure is expected to be difficult, we propose adding a continuous transesophageal echocardiography monitoring using a static 3D/multiplane probe in mid‐esophageal position, with digital remote manipulation of the field of view.

There are of course disadvantages to this approach. Working space in the intervention room is already limited, and continuous monitoring implies longer radiation exposure for the sonographer. We tried to overcome this limitation by using a static probe with an imaging plane that could be remotely rotated toward the target structures. Also, the probe shadow may obstruct the fluoroscopic image, but this was not a real issue in the cases described here.

## 6 CONCLUSION

In selected cases where the complexity of the lead extraction is anticipated to be high, continuous TEE monitoring can be used to increase the success rate of the procedure, prevent or rapidly diagnose complications, or even guide the entire intervention. The use of static 3D/multiplane probes that allow remote manipulation of the field of view can help reduce radiation and improve management of the working space.

## CONFLICT OF INTEREST

All authors declare that they have no competing interests.

## ETHICAL APPROVAL AND CONSENT TO PARTICIPATE

Written informed consent was obtained from the patients for publication of this case series and all accompanying images. A copy of the written consent is available for review by the Editor‐in‐Chief of this journal.

## AVAILABILITY OF DATA AND MATERIAL

The datasets generated and analyzed in the current report are not publicly available due to patient privacy but are available from the corresponding author on reasonable request.

## Supporting information


**Movie S1**. Transesophageal 3D live view of the right cavities (RA: right atrium; RV: right ventricle) in Patient 1,. The ICD lead (yellow arrow) is visualized coursing from the RA through the posteroseptal commissure of the tricuspid valve up to the RV apex.

**Movie S2**. Transesophageal (TEE) 3D live view of the right cavities (RA: right atrium; RV: right ventricle) in Patient 2, as seen from the mid‐esophageal position, during the extraction of the last ventricular lead (arrow). The lead was progressively pulled under hemodynamic, angiographic and TEE monitoring. During pulling there was a near inversion of the RV cavity, which quickly re‐expanded after the lead detached, creating cavitation and microbubbles.

**Movie S3**. Transthoracic apical 4 cavities view at admission of Patient 3. In the right cavities (RA: right atrium; RV: right ventricle) a very weak linear echo (arrow) is visible.

**Movie S4**. Transesophageal 3D live view of the terminal part of the superior vena cava (SVC) in Patient 3, as seen from the mid‐esophageal position, in short axis view. The upper end of the silicone insulation sheath is seen floating freely, without any adherences to the venous wall.

**Movie S5**. Transesophageal biplane live view of the terminal part of the superior vena cava (SVC) in Patient 3, as seen from the mid‐esophageal position, during the extraction of the remaining fragment of the silicone protective tube. The left panel corresponds to a modified bicaval view, with the left atrium (LA) visualized proximal to the probe (in the upper part) and the right atrium (RA) distally (below). The right panel represents a simultaneous perpendicular imaging plane along the dotted line visible in the central panel, focusing in the center on the modified short axis of the SVC. The snare tool (red arrow) is advanced into the superior vena cava and grabs the silicone sheath. Further the extraction tool is pulled back along with the insulation fragment, leaving the SVC free of echoes.
